# Clinical Progression Modes of Crizotinib Failure and Subsequent Management of Advanced Non‐Small Cell Lung Cancer With *ROS*1 Rearrangement

**DOI:** 10.1002/cam4.71592

**Published:** 2026-02-04

**Authors:** Quan‐Quan Tan, Yu‐Qing Chen, Yu‐Er Gao, Ke‐Jun Liu, Zi‐Ji Mao, Ming‐Ying Zheng, Jun‐Wei Su, Jiao Yang, Qing‐Yun Gao, Hua‐Jun Chen, Jin‐Ji Yang

**Affiliations:** ^1^ Guangdong Lung Cancer Institute Guangdong Provincial People's Hospital (Guangdong Academy of Medical Sciences), southern Medical University Guangzhou China; ^2^ Tianjin Medical University Cancer Institute & Hospital National Clinical Research Center for Cancer, Tianjin's Clinical Research Center for Cancer Tianjin China; ^3^ Department of Oncology The Fifth Affiliated Hospital, Southern Medical University Guangzhou China; ^4^ Ezhou Central Hospital Hubei China

**Keywords:** clinical progression mode, NSCLC, *ROS*1, survival benefits

## Abstract

**Background:**

Crizotinib is the classic first‐line treatment for *ROS*1‐rearranged NSCLC. However, data on the clinical progression modes and recommended options for subsequent treatments after crizotinib treatment failure are limited.

**Methods:**

Twenty‐eight patients were categorized into dramatic or gradual/local progression groups. We analyzed the clinical characteristics, survival outcomes, and potential resistance mechanisms in different progression modes.

**Results:**

The median progression‐free survival (mPFS) in the dramatic and gradual/local progression groups was 8.0 and 22.0 months, respectively (*p* < 0.001). The median overall survival (mOS) was 14.2 and 90.3 months in the dramatic progression and gradual/local progression groups, respectively (*p* < 0.001). Among patients with dramatic progression after crizotinib failure, significant differences were shown in median post‐progression overall survival (mpOS) (7.1 vs. 3.6 vs. 1.0 months, *p* = 0.037) and mOS (23.1 vs. 18.3 vs. 10.6 months, *p* = 0.002) across subsequent chemotherapy, targeted therapy, or best supportive care (BSC). *ROS*1 kinase domain point mutations were detected predominantly in the dramatic progression group, while the activation of bypass and downstream pathways occurred in the gradual/local progression group.

**Conclusion:**

The progression modes of *ROS*1 rearrangement may predict survival benefits and provide subsequent treatment strategies in *ROS*1‐rearranged NSCLC.

## Graphical abstract

Survival outcomes in gradual/local progression group were superior to dramatic progression group; survival period of dramatic progression group who received chemotherapy was longer than that of other therapies. *ROS*1 kinase domain mutations were detected predominantly in dramatic progression group, whereas activation of bypass and downstream pathways was found in gradual/local progression group.

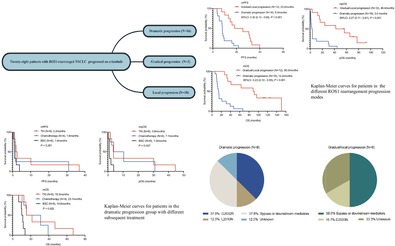

## 1 Introduction

According to the World Health Organization and International Agency for Research on Cancer (WHO‐IARC), lung cancer remains the leading cause of cancer‐related deaths worldwide [1]. Non‐small cell lung cancer (NSCLC) accounts for approximately 85% of all lung cancer cases [2]. The incidence of c‐ros oncogene 1 (*ROS*1) rearrangement is approximately 1%–2% in NSCLC [3, 4, 5]. Crizotinib is a classic first‐line treatment for *ROS*1‐rearranged NSCLC. The PROFILE 1001 study evaluated the efficacy of crizotinib as first‐line therapy in patients with advanced *ROS*1‐rearranged NSCLC. Patients who received crizotinib treatment had a median progression‐free survival (mPFS) of 19.3 months, an objective response rate (ORR) of 72%, and a median overall survival (mOS) of 51.4 months [6]. In the OO12‐01 study, which evaluated the effect of crizotinib in East Asian patients with advanced *ROS*1‐rearranged NSCLC, the mPFS and mOS were 15.9 and 32.5 months, respectively, with an ORR of 71.7% [7]. Several studies have demonstrated the good clinical efficacy of crizotinib in NSCLC [8, 9, 10]. The 2024 Chinese Society of Clinical Oncology guidelines recommend crizotinib and entrectinib as first‐line options for advanced *ROS*1‐rearranged NSCLC [11]. Crizotinib was first approved in China for patients with *ALK*‐rearranged NSCLC in 2013, and then in 2017 it was approved for patients with *ROS*1‐rearranged NSCLC. Meanwhile, entrectinib was approved for patients with *ALK*‐rearranged or *ROS*1‐rearranged NSCLC in 2022. However, lorlatinib has not yet received approval for *ROS*1‐rearranged NSCLC in China. And taking into account factors such as approval, listing status in China, and costs, that often leads to the preferential selection of crizotinib as the first‐line treatment in China [12].

However, the development of drug resistance is an inevitable consequence of drug administration. In the context of acquired resistance, current recommendations include next‐generation tyrosine kinase inhibitors (TKIs) therapy and chemotherapy following crizotinib treatment failure [13]. Several next‐generation *ROS*1 TKIs have shown significant efficacy as first‐line treatment in patients with *ROS*1‐rearranged NSCLC [14, 15, 16, 17]. For example, a clinical study that evaluated lorlatinib for *ROS*1‐rearranged NSCLC showed an ORR of 62% and mPFS of 21.0 months in lorlatinib‐administered TKI‐naïve patients. However, patients previously treated with crizotinib had an ORR of 35% and mPFS of 8.5 months [17]. The efficacy of these TKIs in treating crizotinib‐resistant patients is unsatisfactory compared to other third‐generation *EGFR*‐TKI [18]. Currently, data on treatment options for patients with *ROS*1‐rearranged NSCLC after crizotinib treatment failure are limited. In clinical practice, personalized treatment plans are often based on resistance mechanisms and individual patient condition. Despite previous studies emphasizing the importance of biopsies, real‐world studies have indicated that only a subset of patients undergoes biopsies to definitively identify resistance mechanisms [19, 20]. Therefore, it is important to further explore the clinical progression modes in *ROS*1‐rearranged NSCLC after crizotinib treatment failure to identify the best clinical treatment strategy and improve survival benefits for patients. The clinical progression model for *EGFR* mutations was proposed by Yang et al. [21], as for the treatment background of *ROS*1 rearrangement, it is similar to that of *EGFR* mutation. The standard first‐line treatment of *EGFR* mutation is based on first‐generation TKI, *ROS*1‐rearranged NSCLC is also based on first‐generation TKI, the mPFS of first‐generation *EGFR*‐TKIs was about 9.5–16.0 months [22, 23, 24, 25, 26, 27], the mPFS of first‐generation *ROS*1‐TKIs also was about 11.0–19.3 months [7, 9, 28], the mPFS of first‐line TKI treatment is similar between the two. On the other hand, the resistance mechanisms of *EGFR* mutations [29, 30, 31] and *ROS*1 fusions [32, 33, 34, 35, 36, 37, 38] are similar, with drug resistance target mutations and bypass activation mainly occurring. The definition of acquired resistance to *EGFR*‐TKIs was originally proposed by Jackman et al. [39]. Yang et al. [21] proposed the clinical progression models of *EGFR*‐TKIs resistance based on Jackman et al. [39], the progression model is widely used in clinical practice after Yang et al. [21] proposed it, and many patients with driver genes positive refer to the progression model to choose next treatment strategies after TKI failure. Therefore, based on the above we used the progressive mechanism of *EGFR* mutation for *ROS*1 rearrangement.

This retrospective study simulated the clinical progression model for *EGFR* mutations proposed by Yang et al. [21]. Patients with *ROS*1 rearrangement were grouped into corresponding progression modes after crizotinib treatment failure. Our study aims to explore the clinical progression modes of crizotinib failure in *ROS*l rearrangement NSCLC and the subsequent treatment strategies.

## 2 Methods

### 2.1 Patients and Data Collection

We retrospectively collected data from patients with *ROS*1‐rearranged NSCLC who received crizotinib treatment at the Guangdong Provincial People's Hospital between January 2016 and December 2019. A total of 60 patients were identified with *ROS*1‐rearranged advanced NSCLC, and 28 of them met the following criteria: (1) NSCLC confirmed by the Department of Pathology of Guangdong Provincial People's Hospital, (2) The clinical stage was advanced NSCLC with *ROS*1 rearrangement confirmed by at least one of next generation sequencing (NGS), immunohistochemistry (IHC), and reverse transcription‐polymerase chain reaction (RT‐PCR), (3) Previously treated with crizotinib and had developed crizotinib resistance with sufficient clinical data to analyze the progression modes. However, 32 patients did not meet inclusion criteria and were excluded, of which one patient received traditional Chinese medicine, seven patients received chemotherapy only, four patients received other TKIs, and two patients received crizotinib in combination with other targeted agents, four patients did not develop crizotinib resistance, 11 patients were missing progress assessment data, and three patients developed primary resistance. Hence, 28 patients were included in our study (Figure S1). We retrospectively reviewed the patients' clinical information, pathological types, genetic information, treatment plans, and survival outcomes. This study was approved by the Research Ethics Committee of the Guangdong Provincial People's Hospital and Guangdong Medical Science Institute (Guangzhou, China).

### 2.2 Pathology and IHC

To determine the pathological and immunohistochemical histological subtypes of lung malignancies according to the International Association for the Study of Lung Cancer/American Thoracic Society/European Respiratory Society (IASLC/ATS/ERS) and 2021 WHO classification [40]. Patients in this study Pathological testing and diagnosis were mainly carried out in the Pathology Department of Guangdong Provincial People's Hospital, and the results were independently confirmed by two professional pathologists. Pathological examination uses 4% paraformaldehyde to tissue or pleural effusion sediment, embed it in paraffin, and perform HE staining and immunohistochemistry. Each section is examined under a high‐power field of view of 100 times or 200 times to determine the pathology type.

### 2.3 DNA Sequencing and Data Analysis

The genetic testing of patients before crizotinib treatment and after crizotinib resistance was mainly conducted in the Pathology Department of Guangdong Provincial People's Hospital through IHC, FISH, and RNA testing, and the results were independently confirmed by two professional pathology experts. Patient tissue and liquid biopsy samples were sent to two laboratories, Burning Rock Biotech (Guangzhou, China) and Nanjing Geneseeq Technology Co. Ltd. (Nanjing, China), for NGS testing. The Burning Rock Biotech processed samples using the NextSeq 500 (Illumina) NGS platform, and captured target genes using 168 lung cancer‐related genomes panel or 520 lung cancer‐related genomes panel. NGS was performed on the HiSeq4000 (Illumina) platform or Novaseq6000 NGS platform (Illumina6000) at Nanjing Geneseeq Technology, capturing 425 or 139 lung cancer related genes' panels, respectively. In our study, four patients underwent NGS with 425 panel before and after crizotinib treatment failure, seven patients underwent NGS with 168 panel, three patients underwent NGS with 520 panel, four patients underwent NGS with 139 panel (Table S1).

### 2.4 Response of Evaluation

PFS was defined as the time from treatment initiation to disease progression or death. Overall survival (OS) was defined as the time from treatment initiation to death or last follow‐up. Post‐progression overall survival (pOS) was defined as the time from the start of the next‐line therapy after crizotinib treatment failure to death or the last follow‐up. pOS in the BSC group was defined as the time from the start of the patient's first palliative care program after crizotinib treatment failure to death or the last follow‐up. Crizotinib progression was assessed according to the Response Evaluation Criteria in Solid Tumors version 1.1 (RECIST version 1.1).

### 2.5 Evaluation of *ROS*1 Rearrangement Progression Modes

The progression mode grouping of *ROS*1‐rearranged NSCLC after crizotinib treatment failure strictly followed the clinical modes of EGFR‐TKI failure proposed by Yang et al. [21], and was meticulously evaluated by two clinically experienced doctors. The patients were divided into three groups based on the duration of disease control, evolution of tumor burden, and clinical symptom, regardless of genotype profile, according to Yang et al. [21]. Duration of disease control was defined as the interval between crizotinib treatment and the first documentation of progressive disease. Evolution of tumor burden was represented by the volume doubling time of target lesions and progressive involvement in nontarget lesions between the two latest consecutive assessments. Progression in nontarget lesions was defined as progression of preexisting lesions, progression due to new lesions in the thoracic cavity, new lesions beyond the thoracic cavity, or new malignant effusion, each progression was scored as 1. Quantification of progressive involvement in nontarget lesions was expressed as a score of 1–4 [21, 41]. Clinical symptom was quantified based on six items: cough, hemoptysis, chest pain, fever, dyspnea and metastatic lesion related symptom, on the symptom score, a score of 0 is asymptomatic status, 1 is stable with pre‐existing item, and 2 is a deterioration of any pre‐existing item or new item [21, 42, 43]. Patients in the dramatic progression group demonstrated the following criteria: disease control lasting ≥ 3 months with crizotinib treatment [20] compared with previous assessment, rapid progression of multiple target lesions, or progressive involvement of nontarget lesions with a score > 2 [19, 21], and symptom scored 2 [21, 42, 43]. Patients in the gradual progression group met the following criteria: disease control lasting ≥ 6 months with crizotinib treatment [44, 45] compared with previous assessment, no significant increment of tumor burden and progressive involvement of nontarget lesions with a score ≤ 2 [19, 41], and symptom scored ≤ 1 [21, 42, 43]. Patients in the local progression group met the following criteria: disease control lasting ≥ 3 months with crizotinib treatment [20], PD due to solitary extracranial lesion or limitation in intracranial lesions [46, 47, 48, 49], and symptom scored ≤ 1 [21, 42, 43] (Table 1).

**TABLE 1:** Evaluative criteria for progression modes in *ROS*1 rearranged NSCLC after crizotinib failure.

Dramatic progression	Gradual progression	Local progression
(1) Disease control lasting ≥ 3 months with crizotinib treatment [20]	(1) Disease control lasting ≥ 6 months with crizotinib treatment [44, 45]	(1) Disease control lasting ≥ 3 months with crizotinib treatment [20]
(2) Compared with the previous assessment, rapid progression of multiple target lesions, or progressive involvement of nontarget lesions with a score > 2 [19, 41]	(2) Compared with the previous assessment, no significant increment of tumor burden and progressive involvement of nontarget lesions with a score ≤ 2 [19, 41]	(2) PD due to solitary extracranial lesion or limitation in intracranial lesions (covered by a radiation field) [46, 47, 48, 49]
(3) Symptom scored 2 [21, 42, 43]	(3) Symptom scored ≤ 1 [21, 42, 43]	(3) Symptom scored ≤ 1 [21, 42, 43]

Abbreviations: NSCLC, non‐small cell lung cancer; PD, progressive disease.

### 2.6 Statistical Analysis

Categorical variables are expressed as numbers (*N*) and percentages (%), and were compared using the Chi‐square or Fisher's exact test. PFS, OS, and pOS were calculated using Kaplan–Meier curves, and log‐rank tests were used to compare the differences between variables. All statistical analyses were performed using GraphPad Prism (GraphPad Software 9.0), and IBM SPSS version 26 (SPSS Statistics V26, IBM Corporation, Somers, New York).

## 3 Results

### 3.1 Baseline Characteristics of Patients

According to the inclusion and exclusion criteria, 28 patients were selected for grouping between January 2016 and December 2019 (Figure S1). Among these, 57.1% (16/28) were included in the dramatic, 35.7% (10/28) in the local, and 7.1% (2/28) in the gradual progression groups (Table 2). We merged the gradual and local progression groups for analogous survival. In the dramatic progression group, 87.5% of patients were aged ≤ 60 years, 56.3% were female, 93.8% were adenocarcinoma, 81.3% had no brain metastases before receiving crizotinib treatment, and 37.5% received crizotinib treatment at the first line. In the gradual/local progression group, 91.7% of patients were aged ≤ 60 years, 66.7% were female, all 12 patients were adenocarcinoma, 91.7% of patients had no brain metastases before crizotinib treatment, and 75.0% of patients received crizotinib treatment at the first line. There were no differences between the dramatic and gradual/local progression groups in terms of age, gender, smoking history, pathological type, stage, brain metastasis, Eastern Cooperative Oncology Group Performance Status (ECOG PS) score, detection method, or treatment line (Table 2).

**TABLE 2:** Baseline patient characteristics in the different progression modes (*N* = 28).

	Dramatic progression (16, *n*, %)	Gradual/local progression (12, *n*, %)	*p*
Age (years)			
≤ 60	14 (87.5)	11 (91.7)	1.000
> 60	2 (12.5)	1 (8.3)	
Gender			
Female	9 (56.3)	8 (66.7)	0.705
Male	7 (43.8)	4 (33.3)	
Smoking history			
Never	15 (93.8)	11 (91.7)	1.000
Smoker	1 (6.3)	1 (8.3)	
Histology			
Adenocarcinoma	15 (93.8)	12 (100.0)	1.000
Large‐cell carcinoma	1 (6.3)	0 (0.0)	
Stage			
IVA	8 (50.0)	3 (25.0)	0.253
IVB	8 (50.0)	9 (75.0)	
Brain metastasis			
Yes	3 (18.8)	1 (8.3)	0.613
No	13 (81.3)	11 (91.7)	
ECOG PS score			
1	15 (93.8)	11 (91.7)	1.000
0	1 (6.3)	1 (8.3)	
Line of crizotinib			
1st	6 (37.5)	9 (75.0)	0.067
≥ 2nd	10 (62.5)	3 (25.0)	
Fusion partner			
CD74	6 (37.5)	7 (58.3)	0.496
Non‐CD74	6 (37.5)	2 (16.7)	
Unknown	4 (25.0)	3 (25.0)	
The text method			
Others	6 (37.5)	5 (41.7)	1.000
NGS	9 (56.3)	6 (50.0)	
Unknown	1 (6.3)	1 (8.3)	
Subsequent treatment			
TKI	6 (37.5)	9 (75.0)	0.050
Chemo	4 (25.0)	3 (25.0)	
BSC	6 (37.5)	0 (0.0)	

Abbreviations: BSC, best supportive care; ECOG PS, Eastern Cooperative Oncology Group Performance Status; NGS, next generation sequencing; TKI, tyrosine kinase inhibitor.

### 3.2 Survival of Different *ROS*1 Rearrangement Progression Modes

**FIGURE 1:**
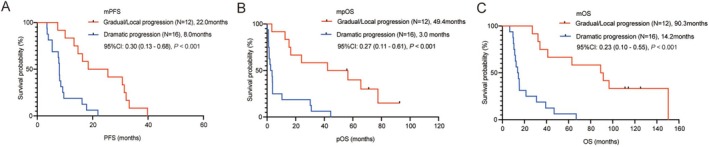
Kaplan‐Meier curves for patients in the different *ROS*1 rearrangement progression modes. Progression‐free survival (A), post‐progression overall survival (B), and overall survival (C) of patients in different *ROS*1 rearrangement progression groups. PFS, progression‐free survival; pOS, post‐progression overall survival; OS, overall survival.

As of September 15, 2025, 10.7% (3/28) of the patients are still alive. The mPFS of the gradual/local progression group was longer than that of the dramatic progression group (22.0 vs. 8.0 months, HR = 0.30, 95% confidence interval [CI]: 0.13–0.68, *p* < 0.001) (Figure 1A). The mpOS of the gradual/local progression group was superior to that of the dramatic progression group (49.4 vs. 3.0 months, HR = 0.27, 95% CI: 0.11–0.61, *p* < 0.001) (Figure 1B). Patients in the gradual/local progression group had significantly longer OS than those in the dramatic progression group (90.3 vs. 14.2 months, HR = 0.23, 95% CI: 0.10–0.55, *p* < 0.001) (Figure 1C).

### 3.3 Subsequent Treatments of Different *ROS*1 Rearrangement Progression Modes

**FIGURE 2:**
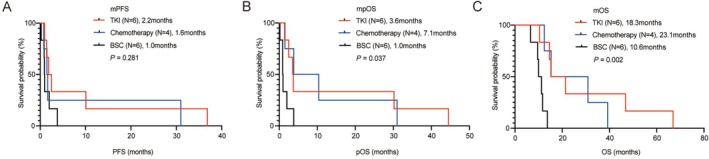
Kaplan‐Meier curves for patients in the dramatic progression group with different subsequent treatments. Progression‐free survival (A), post‐progression overall survival (B), and overall survival (C) of patients in the dramatic progression group with different subsequent treatments after crizotinib failure. OS, overall survival; PFS, progression‐free survival; pOS, post‐progression overall survival.

In the dramatic progression group, the post‐crizotinib treatment regimens were TKI in 37.5% (6/16) of patients, chemotherapy in 25% (4/16), and best supportive care (BSC) in the remaining 37.5% (6/16) (Table S1). No significant difference was noted in mPFS (2.2 vs. 1.6 vs. 1.0 months, *p* = 0.281) (Figure 2A) among patients receiving next‐line treatment with TKI, chemotherapy, or BSC after crizotinib treatment failure in the dramatic progression group. However, the mpOS (*p* = 0.037; Figure 2B) and mOS (*p* = 0.002; Figure 2C) were significantly different in patients who received the different subsequent treatments in the dramatic progression group. The mpOS was longer in the chemotherapy (7.1 vs. 1.0 months, HR = 0.34, 95% CI: 0.09–1.26, *p* = 0.040) and TKI (3.6 vs. 1.0 months, HR = 0.37, 95% CI: 0.10–1.32, *p* = 0.051) groups, compared to that in the BSC group, but the differences were borderline significant (Figure 2C). The mpOS of the TKI group was longer than that of the chemotherapy group (3.6 vs. 7.1 months, HR = 1.03, 95% CI: 0.29–3.65, *p* = 0.956) (Figure 2C). The chemotherapy group demonstrated a longer mOS compared to the BSC group (23.1 vs. 10.6 months, HR = 0.27, 95% CI: 0.07–1.06, *p* = 0.012), although the mOS of the TKI group was shorter than that of the chemotherapy group (18.3 vs. 23.1 months, HR = 0.77, 95% CI: 0.21–2.84, *p* = 0.656). The mOS of the TKI group was longer than that of the BSC group (18.3 vs. 10.6 months, HR = 0.27, 95% CI: 0.07–1.07, *p* = 0.0049) (Figure 2B).

In the gradual/local progression group, 75% (9/12) of the patients received TKI therapy, while the remaining 25% (3/12) received chemotherapy (Table S1). Patients with gradual/local progression primarily received TKI treatment or chemotherapy after crizotinib treatment failure. No significant difference was noted in mPFS between those that received TKI or chemotherapy (6.2 vs. 7.6 months, HR = 1.39, 95% CI: 0.40–4.84, *p* = 0.618). However, patients treated with TKIs had longer mpOS (56.5 vs. 24.1 months, HR = 0.68, 95% CI: 0.15–3.06, *p* = 0.577) and OS (91.4 vs. 63.0 months, HR = 0.44, 95% CI: 0.08–2.40, *p* = 0.220) than those treated with chemotherapy. In addition, 25.0% (3/12) patients did not reach the event endpoint at the last follow‐up (Figure S2).

### 3.4 Potential Resistance Mechanisms Underlying the Progression Modes

**FIGURE 3:**
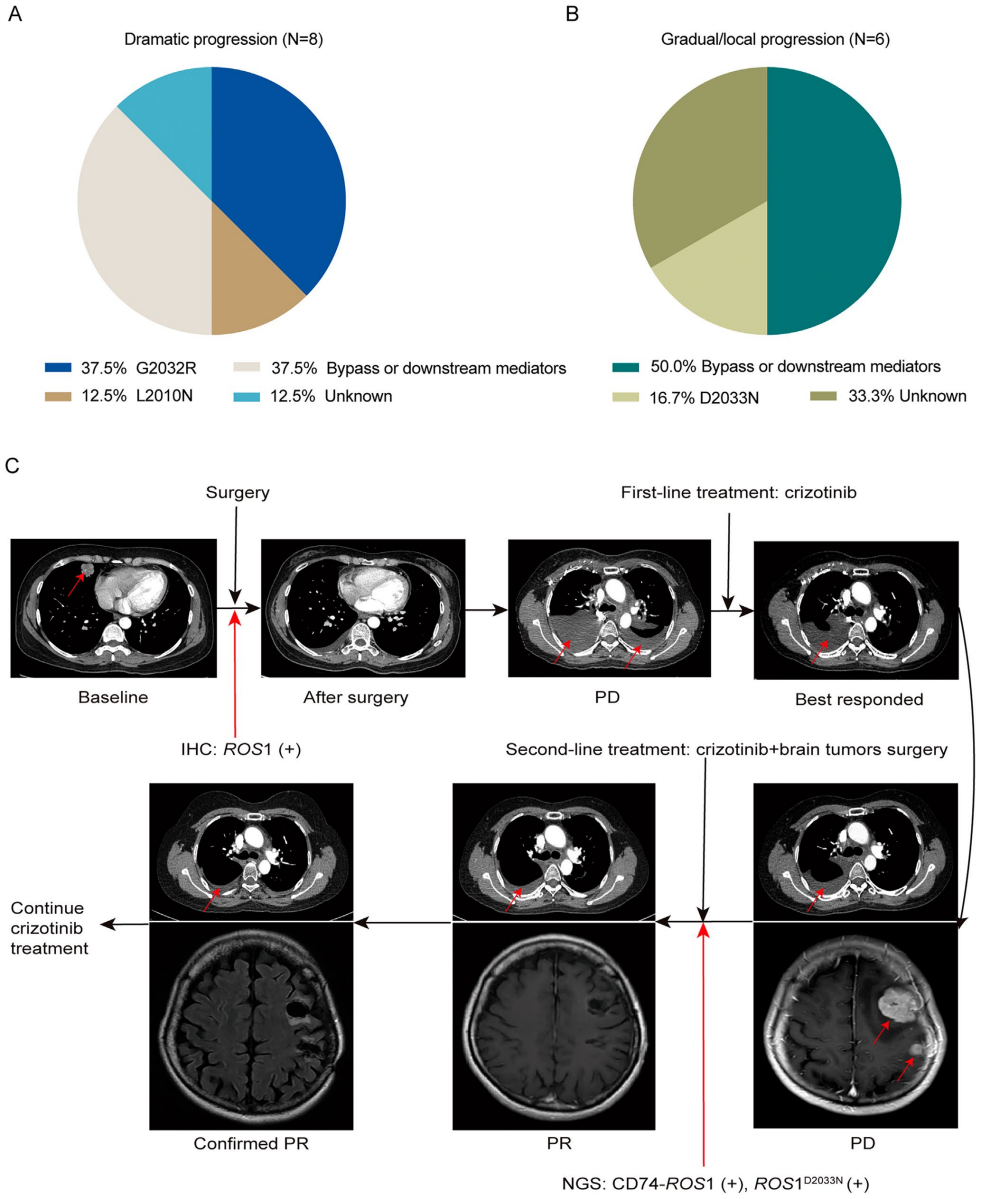
Resistance mechanisms in *ROS*1 rearrangement progression modes and a typical case presentation. (A) Distribution of resistance mechanisms in the dramatic progression group. (B) Distribution of resistance mechanisms in the gradual/local progression group. (C) A case presentation from the local progression group.

In the present study, among the 16 patients in the dramatic progression group, five did not undergo re‐biopsy after crizotinib treatment, whereas 11 patients did. Among these patients, one underwent RT‐PCR and 10 underwent NGS. The NGS results were not available for two patients. Seven patients underwent NGS of matched specimens prior to crizotinib treatment, whereas six patients did so both before and after crizotinib treatment. Among the eight patients who underwent NGS, 37.5% (3/8) acquired *ROS*1^G2032R^ after crizotinib treatment. In addition, NGS revealed that 12.5% (1/8) acquired *ROS*1^L2010M^ after crizotinib treatment failure and 37.5% (3/8) exhibited activation of the RAS/RAF/MEK, PI3K, or JAK/STAT signaling pathways (Figure 3A). The resistance mechanism was unclear in 12.5% (1/8) of patients.

Among the 12 patients in the gradual/local progression group, 50.0% (6/12) underwent a re‐biopsy after crizotinib treatment failure and 16.7% (1/6) of these were detected with *ROS*1^D2033N^. Moreover, 50.0% (3/6) of the re‐biopsied patients showed activation of bypass tracks (Figure 3B). In one patient in this subgroup, the *ROS*1 rearrangement disappeared after progression, and only the TP53 mutation remained, which may be related to tumor heterogeneity, whereas no clear resistance mechanism was noted in two patients. In addition, none of the patients who underwent re‐biopsy after disease progression in our study had pathological phenotypic changes (Table S2). No significant differences in the resistance mechanisms were noted between the two groups (*p* = 0.5).

A typical case presentation from the local progression group. Figure 3C illustrates the diagnostic and treatment course for a case with local progression. A 48‐year‐old woman was initially diagnosed with early stage right lower lung cancer and underwent surgical treatment, which revealed an adenocarcinoma of the lung with *ROS*1 rearrangement. The patient received adjuvant cisplatin and etoposide treatment and synchronous mediastinal lymph node radiotherapy. Approximately 11.3 months after surgery (disease‐free survival [DFS] of 11.3 months), bilateral malignant pleural effusions were observed, and the patient was evaluated for PD. Due to the detection of *ROS*1 rearrangement at baseline, the patient received crizotinib treatment. However, after 32.1 months, multiple brain metastases were detected, whereas the pulmonary lesions remained stable. The patient did not present with cough, hemoptysis, dizziness, or headache. Therefore, the patient was diagnosed with local progression. Subsequently, the patient underwent brain tumors surgery, which revealed an adenocarcinoma on histopathological examination. The NGS results of the brain tissue revealed not only CD74‐*ROS*1 rearrangement but also *ROS*1 D2033N mutation. However, the patient continued to receive crizotinib treatment after the surgery and achieved PR with a PFS of 92.7 months. The patient continued crizotinib therapy at follow‐up.

## 4 Discussion

Our study investigated the clinical progression modes for *ROS*1‐rearranged NSCLC after crizotinib resistance and subsequent treatment strategies. The clinical progression modes in *ROS*1‐rearranged NSCLC after crizotinib treatment failure provided real‐world survival data on disease progression modes, subsequent treatment strategies, and potential resistance mechanisms. To our knowledge, this is the first study to examine the clinical progression of *ROS*1 rearrangement following crizotinib treatment failure.

In our study, *ROS*1 rearrangement was detected in young nonsmoking women with lung adenocarcinoma, which is consistent with previous studies [4, 50]. Our survival outcomes of the progression modes in *ROS*1 rearranged NSCLC were similar to those of EGFR‐TKI failure [21]. The survival outcomes in the gradual/local progression group were superior to those in the dramatic progression group. Therefore, to some extent, the differences in survival outcomes between *ROS*1 rearrangement clinical progression modes provide real‐world data for predicting patient prognosis and survival outcomes. Regarding the choice of subsequent treatment, the survival period of patients in the dramatic progression group who received chemotherapy was longer than that of patients who received TKI or BSC therapies. This is consistent with the results of Yang et al. [21], and suggests that these progression modes may also be applicable to patients with *ROS*1 rearranged NSCLC. Patients with gradual/local progression mainly present with progressive involvement of nontarget lesions, solitary extracranial lesion, or limitation in intracranial lesions. A previous study demonstrated that patients with oncogene‐addicted NSCLC who experienced central nervous system progression and/or oligoprogression benefited from TKI therapy in combination with local treatment [51]. Additionally, in our study, more than half of the patients received TKI therapy in combination with local therapy, and the OS of patients receiving TKIs therapy tended to be longer than that of patients receiving chemotherapy in the gradual/local progression group. In terms of the survival trend of the overall population, patients with dramatic disease progression who received chemotherapy, and those with gradual/local disease progression who received TKI treatment may have better survival benefits.

TKI resistance remains a major challenge that needs to be addressed. The resistance mechanisms of *ROS*1 rearrangement have been categorized into three primary types: (1) *ROS*1 kinase domain point mutations, including mutations such as G2032R, D2033N, L1951R, S1986F, S1986Y and L2026M [32, 33, 34], (2) activation of bypass tracks and aberrations in downstream pathways, such as activation of the *EGFR*, *KRAS*, RAS/RAF/MEK, PI3K, and JAK/STAT pathways [34, 35, 36, 37, 38], (3) the alterations in pathological phenotype [52, 53]. In our study, we found that different resistance mechanisms would emerge depending on the specific pattern of the *ROS*1 rearrangement process. *ROS*1 kinase domain point mutations primarily occurred in the dramatic progression group, whereas the activation of bypass tracks and downstream pathways mainly occurred in the gradual/local progression group. Notably, the rate of *ROS*1 kinase domain point mutations was higher in the dramatic progression group than in the gradual/local progression group, *ROS*1^G2032R^ remained the most common mechanism of resistance to crizotinib in the dramatic progression group, and its occurrence was close to that of the general population receiving crizotinib treatment. Previous studies reported that the incidence of *ROS*1^G2032R^ mutation in patients who are resistant to crizotinib therapy is approximately 38%–53% [53, 54, 55]. However, some patients did not have clear gene variants of resistance, which suggests the existence of non‐genomic factors that may lead to TKI resistance. The treatment of crizotinib resistance in *ROS*1‐rearranged NSCLC is mainly based on the traditional molecular mechanism of crizotinib resistance; for example, there is a clear change in the resistance site [55]. Our clinical progression models have taken the molecular resistance mechanism into consideration and have a reference value for patients who do not carry driver genes. Therefore, further distinguishing between the different resistance mechanisms would help in evaluating the choice of second‐line treatment options.

However, there are some limitations in our study. Firstly, due to the low mutation rate of *ROS*1 rearrangement in NSCLC, our study had a small sample size that could not be validated, making it impossible to assign groups based on the statistical methods. In addition, we retrospectively obtained patient data from past medical records, and some clinical information was unavailable. Additionally, not every patient underwent a re‐biopsy after crizotinib treatment failure, which limited further detailed analyses of resistance mechanisms. Therefore, further studies with larger sample sizes are needed to further examine resistance‐related mechanisms.

## 5 Conclusion

This study provides real‐world survival data and potential resistance mechanisms associated with different *ROS*1 rearrangement clinical progression modes. Clinical progression modes of *ROS*1 rearranged NSCLC may help in predicting the prognosis and formulating subsequent treatment plans. Further studies are warranted to examine the clinical progression modes and mechanisms of resistance after crizotinib treatment failure.

## Author Contributions

**Quan‐Quan Tan:** conceptualization, data curation, formal analysis, investigation, methodology, visualization, writing – original draft, writing – review and editing. **Yu‐Qing Chen:** investigation, methodology, writing – review and editing. **Yu‐Er Gao:** investigation, methodology, writing – review and editing. **Ke‐Jun Liu:** investigation, writing – review and editing. **Zi‐Ji Mao:** investigation, writing – review and editing. **Ming‐Ying Zheng:** investigation, writing – review and editing. **Jun‐Wei Su:** investigation, writing – review and editing. **Jiao Yang:** investigation, writing – review and editing. **Qing‐Yun Gao:** investigation, writing – review and editing. **Hua‐Jun Chen:** conceptualization, investigation, methodology, writing – review and editing. **Jin‐Ji Yang:** funding acquisition, conceptualization, investigation, methodology, writing – review and editing.

## Disclosure

The authors have nothing to report.

## Ethics Statement

The present study was approved by the Ethics Committee of Guangdong Provincial People's Hospital (Guangzhou, China).

## Consent

The authors thank all the patients who participated in this study. All patients included in the study gave written informed consent to participate in the study.

## Conflicts of Interest

The authors declare no conflicts of interest.

## Supporting information


**Figure S1:** Flow chart illustrating the study design.

**Figure S2:** Kaplan–Meier curves for patients in gradual/local progression group with different subsequent treatments after crizotinib failure. Progression‐free survival (A), post‐progression overall survival (B), and overall survival (C) of patients in gradual/local progression group with different subsequent treatments after crizotinib failure.

**Table S1:** Treatment regimen of patients in the *ROS*1 rearrangement progression modes.

**Table S2:** Gene profile of patients in different *ROS*1 rearrangement progression groups.

## Data Availability

The datasets used and analyzed during the current study are available from the corresponding author on reasonable request.
