# Supplementary figures and images for: Clinical Progression Modes of Crizotinib Failure and Subsequent Management of Advanced Non‐Small Cell Lung Cancer With ROS1 Rearrangement
Source: Cancer Med. 2026 Feb 4;15(2):e71592. doi: 10.1002/cam4.71592 (PMC12872283; doi:10.1002/cam4.71592)

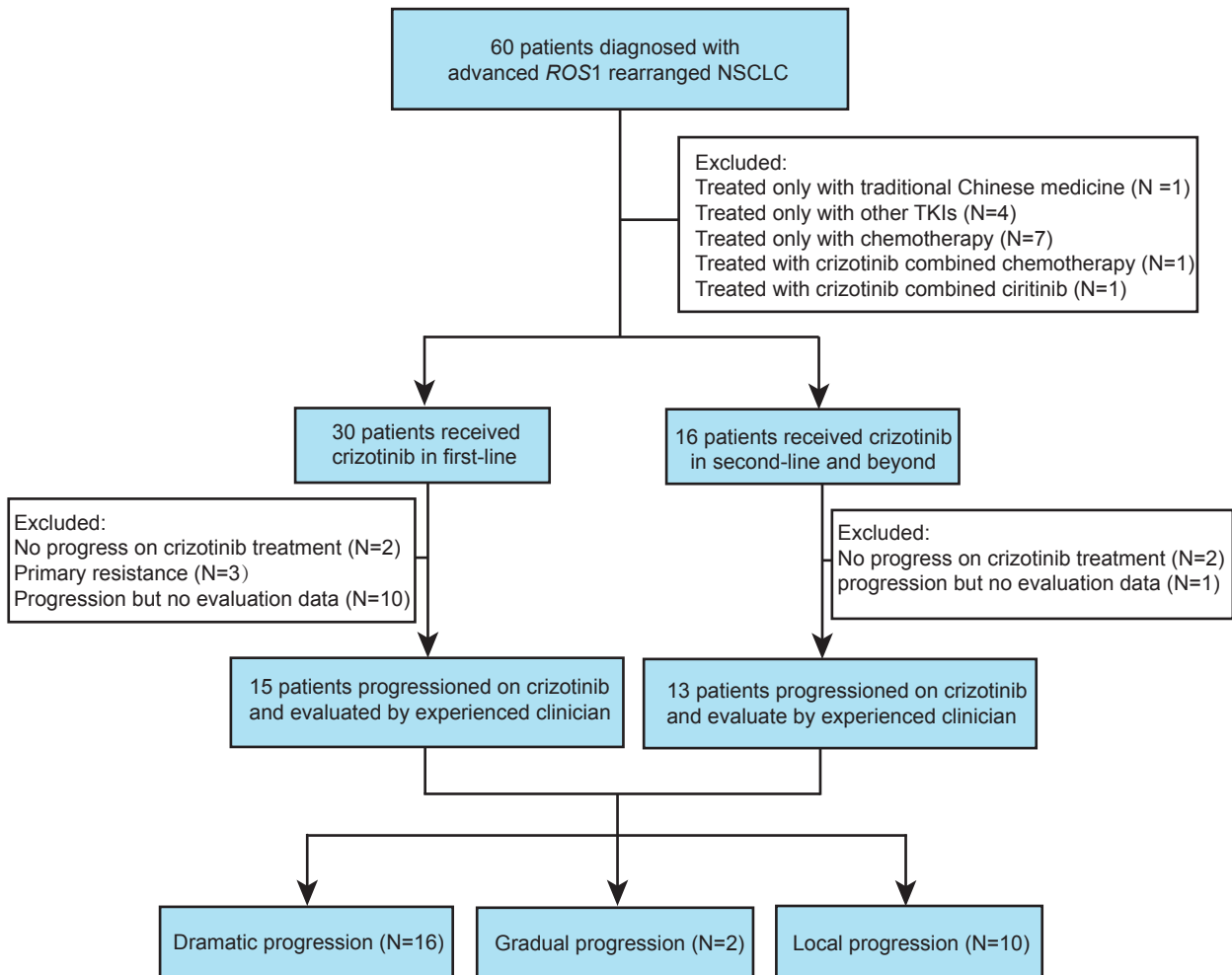

**Supplementary Fig. 1** Flow chart illustrating the study design.

Supplement: Supplementary file 1 — Figure S1: Flow chart illustrating the study design. [file CAM4-15-e71592-s004.pdf]

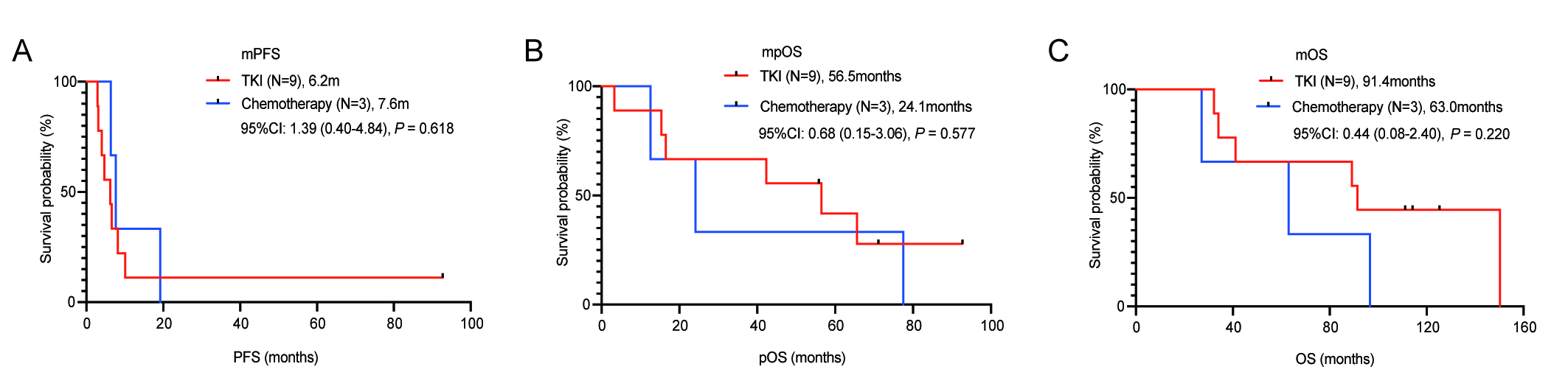

Supplement: Supplementary file 2 — Figure S2: Kaplan–Meier curves for patients in gradual/local progression group with different subsequent treatments after crizotinib failure. Progression‐free survival (A), post‐progression overall survival (B), and overall survival (C) of patients in gradual/local progression group with different subsequent treatments after crizotinib failure. [file CAM4-15-e71592-s002.tif]
