# Supplementary material for: Clinical Progression Modes of Crizotinib Failure and Subsequent Management of Advanced Non‐Small Cell Lung Cancer With ROS1 Rearrangement
Source: Cancer Med. 2026 Feb 4;15(2):e71592. doi: 10.1002/cam4.71592 (PMC12872283; doi:10.1002/cam4.71592)
Supplement: Supplementary file 3 — Table S1: Treatment regimen of patients in the ROS1 rearrangement progression modes. [file CAM4-15-e71592-s001.docx]

**Supplementary Table 1**  Treatment regimen of patients in the *ROS*1 rearrangement progression modes

| Patient ID | Progression mode | Sex | Age  (years) | Fusion partner | Crizotinib treatment  (treatment-line/PFS) | PS  (After crizotinib) | Subsequent therapy after crizotinib treatment failure  (treatment-line/PFS) |
| --- | --- | --- | --- | --- | --- | --- | --- |
| 1 | Dramatic progression | F | 31 | CD74 | 1th/16.2m | 1 | Lorlatinib (10.1m) |
| 2^‡^ | Dramatic progression | M | 47 | CD74 | 2nd/5.4m | 1 | Alectinib (1.4m) |
| 3^‡^ | Dramatic progression | M | 46 | CD74 | 1th/9.2m | 1 | BSC (0.2m) |
| 4 | Dramatic progression | F | 48 | EZR | 2nd/7.8m | 1 | Bevacizumab + Nab-paclitaxel（0.8m） |
| 5 | Dramatic progression | F | 61 | EZR | 2nd/8.0m | 1 | BSC (0.9m) |
| 6 | Dramatic progression | M | 29 | SDC4 | 2nd/17.8m | 1 | Crizotinib + Local therapy (2.5m) |
| 7 | Dramatic progression | F | 51 | SDC4 | 1th/7.6m | 1 | BSC (2.0m) |
| 8 | Dramatic progression | F | 29 | SLC34A2 | 2nd/8.1m | 1 | BSC (1.0m) |
| 9 | Dramatic progression | M | 42 | SLC34A2 | 2nd/8.5m | 1 | Pemetrexed + Carboplatin (1.5m) |
| 10 | Dramatic progression | F | 62 | CD74 | 1th/21.9m | 1 | Lorlatinib (36.8m) |
| 11 | Dramatic progression | M | 58 | *ROS*1(+) | 2nd/5.4m | 1 | BSC (3.8m) |
| 12 | Dramatic progression | M | 44 | *ROS*1(+) | 2nd/3.6m | 2 | Crizotinib (1.9m) |
| 13 | Dramatic progression | F | 33 | *ROS*1(+) | 2nd/3.5m | 2 | BSC (0.9m) |
| 14^‡^ | Dramatic progression | F | 52 | CD74 | 2nd/4.0m | 1 | ABCP (1.7m) |
| 15 | Dramatic progression | F | 56 | CD74 | 1th/9.6m | 2 | Foritinib (1.0m) |
| 16 | Dramatic progression | M | 44 | *ROS*1(+) | 1th/8.0m | 1 | Pemetrexed + Bevacizumab (31.0m) |
| 17 | Gradual/local progression | F | 46 | CD74 | 1th/39.8m | 1 | Brigatinib (8.2m) |
| 18 | Gradual/local progression | F | 42 | CD74 | 6th/30.7m | 1 | Foritinib (4.6m) |
| 19^‡^ | Gradual/local progression | F | 48 | CD74 | 1th/32.1m | 1 | Crizotinib + Local therapy^†^ (92.7m) |
| 20 | Gradual/local progression | F | 58 | CD74 | 4th/25.5m | 1 | Ceritinib +Local therapy (4.0m) |
| 21 | Gradual/local progression | M | 56 | EZR | 1th/14.7m | 1 | Pemetrexed + Carboplatin + Bevacizumab (7.6m) |
| 22 | Gradual/local progression | F | 30 | SDC4 | 1th/10.1m | 1 | Cabozantinib + Local therapy (6.2m) |
| 23 | Gradual/local progression | M | 53 | *ROS*1(+) | 1th/31.4m | 1 | Pemetrexed + Carboplatin + Bevacizumab (19.2m) |
| 24 | Gradual/local progression | M | 51 | ROS1(+) | 1th/16.3m | 1 | Crizotinib + Local therapy (10.1m) |
| 25 | Gradual/local progression | F | 51 | CD74 | 2nd/7.4m | 1 | Pemetrexed + Carboplatin + Bevacizumab (6.3m) |
| 26 | Gradual/local progression | M | 64 | *ROS*1(+) | 1th/18.4m | 1 | Ceritinib (6.6m) |
| 27 | Gradual/local progression | F | 56 | CD74 | 1th/33.2m | 1 | Foritinib (2.9m) |
| 28 | Gradual/local progression | F | 48 | CD74 | 1th/13.3m | 2 | Crizotinib + Local therapy (3.1m) |

Abbreviation: F, female; M, male; *ROS1*(+), c-ros oncogene 1 fusion positive; ADC, adenocarcinoma; LCC, large cell carcinoma; ECOG PS, Eastern Cooperative Oncology Group Performance Status; NGS, next generation sequencing; BSC, best supportive care; ABCP, atezolizumab + bevacizumab + carboplatin + paclitaxel; m, months; †, the treatment regimen was continued at the end of follow-up; ‡, patient ID of 19 with *ROS*1^p.D2033N^ mutation after crizotinib failure, patient ID of 2 with *ROS*1^p.G2032R^ mutation after crizotinib failure, patient ID of 3 with *ROS*1^p.G2032R^ mutation after crizotinib failure, patient ID of with14 with ROS1^p.G2032R^ mutation after crizotinib failure.
