# Supplementary material for: Clinical Progression Modes of Crizotinib Failure and Subsequent Management of Advanced Non‐Small Cell Lung Cancer With ROS1 Rearrangement
Source: Cancer Med. 2026 Feb 4;15(2):e71592. doi: 10.1002/cam4.71592 (PMC12872283; doi:10.1002/cam4.71592)
Supplement: Supplementary file 4 — Table S2: Gene profile of patients in different ROS1 rearrangement progression groups. [file CAM4-15-e71592-s003.docx]

**Supplementary Table 2** Gene profile of patients in different *ROS*1 progression groups

| Patient ID | Progression mode | Before crizotinib treatment | | |  | After crizotinib treatment | | |
| --- | --- | --- | --- | --- | --- | --- | --- | --- |
|  |  | Method  (NGS assay) | Biopsy site | Gene profile |  | Method  (NGS assay) | Biopsy site | Gene profile |
| 1 | Gradual/local progression | NGS  (168 panel) | Lung | CD74-ROS1(fusion), CTNNB1（p.G34V）, LRP1B（p.G4297R）, MAP3K1（p.P15A） |  | Non-biopsy | Non-biopsy | Non-biopsy |
| 2 | Gradual/local progression | NGS  (168 panel) | Lung | CD74-ROS1（fusion）, STAG2(p.T1151I), ALK（p.P158=）, PRKDC(p.D1005Y), NTRK3(p.L209H) |  | NGS  (168 panel) | Lung | APC (fusion), CD74 (fusion, ANKRD17-CD74), CD74-ROS1(fusion) |
| 3 | Gradual/local progression | IHC | Lung | ROS1(+) |  | NGS  (425panel) | Brain | ERBB4(p.F478V), GATA4 (p.T366M), HNF1A (p.G319S), MAP2K2 (p.P10L), PGR (p.E695K), ROS1-CD74(fusion), ROS1(p.D2033N), SRC (p.A31V), STAG2(p.E750K), TNFRSF11A (p.E600K), TSC2(p.S1433L) |
| 4 | Gradual/local progression | NGS  (425 panel) | Plasma | ROS1(+) |  | NGS  (425panel) | Brain | ROS1-CD74(fusion), BRCA1(fusion), CDKN2A(fusion), TP53(p.P152L), LRP1B（Del）, IGR (upstream ZNF32)~RET:exon14 |
| 5 | Gradual/local progression | NGS  (168 panel) | Plasma | KCNA3-ROS1(fusion), EZR-ROS1(fusion), TP53(p.S183*) |  | NGS  (168 panel) | Chest nodule | PTK2(Amp), MYC(Amp), EZR-ROS1(fusion), KCNA3-ROS1(fusion), SPEN (p.E376D), ATM (missense_variant), HDAC4(missense_variant), XPO1, GNAS (p.S443A), BACH1(p.C279S), WRN (p.Q1239E), PRKDC (p. L293=), ABL1(p.F283L), TP53(p.S183*), MAP3K13(p.D875N), NTRK3(p.S492*) |
| 6 | Gradual/local progression | NGS  (168 panel) | Lymph nodes | ROS1(+) |  | Non-biopsy | Non-biopsy | Non-biopsy |
| 7 | Gradual/local progression | IHC | Lymph nodes | ROS1(+) |  | NGS  (168 panel) | Brain | TP53(p. Pro72Arg) |
| 8 | Gradual/local progression | FISH | Pleura | ROS1(+) |  | Non-biopsy | Non-biopsy | Non-biopsy |
| 9 | Gradual/local progression | IHC | Lung | ROS1(+) |  | Non-biopsy | Non-biopsy | Non-biopsy |
| 10 | Gradual/local progression | NA | Lung | ROS1(+) |  | NGS  (NA) | Liver | ROS1(+) |
| 11 | Gradual/local progression | NGS  (520 panel) | Lung | JAK1(fusion), CD74-ROS1(fusion), MIR5685-ROS1(fusion), TAF1(p. A19fs), JAK1(p.V315=), BAP1(p.V171fs), HIST1H3G(p.G45D), KDM5C(p.Q96*), SMAD4(p.D351V), ROS1(p.K1996R) |  | Non-biopsy | Non-biopsy | Non-biopsy |
| 12 | Gradual/local progression | FISH | Lymph nodes | ROS1(+) |  | NGS  (520 panel) | Cerebrospinal fluid | CD74-ROS1(fusion), CTNNB1(p.S33A) |
| 13 | Dramatic progression | NGS  (168 panel) | Lymph nodes | CD74-ROS1(fusion), OR5L2(p.C107fs) |  | NGS  (168 panel) | Cerebrospinal fluid | CD74-ROS1(fusion), TRPC5(p. K8=), |
| 14 | Dramatic progression | NGS  (139 panel) | Lung | DPYD (p.I543V), UGT1A1(p.G71R), GSTT1(Del), NQO1(p.P187S), CD74-ROS1(fusion), BIM(Del) |  | NGS  (139 panel) | Lung | BCL2L11(Del), GSTT1(Del), NQO1(p.P187S), UGT1A1(p.G71R), ROS1(p.G2032R), DPYD (p.I543V), CD74-ROS1(fusion) |
| 15 | Dramatic progression | NGS  (139 panel) | Lung | CD74-ROS1(fusion), FGFR3(p.V323F), GNAQ (p.I190V), TP53(p.R283_E286del) |  | NGS  (139 panel) | Lung | PIK3R1(amp), STK11(amp), CD74-ROS1(fusion), BRINP3(p.P594T), APC(p.D256N), TP53(p.R283_E286del), ROS1（p.G2032R） |
| 16 | Dramatic progression | FISH | Chest nodule | ROS1(+) |  | NGS  (139 panel) | Liver | CDK4(p.V54I), TP53(Del), ROS1-CCDC28A(fusion) , EZR-ROS1(fusion), STK11(p.F354L) |
| 17 | Dramatic progression | NGS  (520 panel) | Lymph nodes | DCBLD1-ROS1(fusion), EZR-ROS1(fusion), INSR (p. P269=), CREBBP (p.P248A), LRP1B (p.A3093T), IKZF1(p.M518K) |  | NGS  (520 panel) | Lung | FGFR1(amp), FGFR3(amp), DCBLD1-ROS1(fusion), EZR-ROS1(fusion), INSR (p. P269=), CREBBP (p.P248A), LRP1B (p.A3093T), IKZF1(p.M518K), DNMT3A (p.L639F), TP53(p.S183*) |
| 18 | Dramatic progression | NGS  (425 panel) | Lymph nodes | GATA3(p.P218H), STAT3(p.P218H), TP53(p.S127F), MYH9(p.1466del), NOTCH1(Del), SMARCA4(Del), SDC4-ROS1(fusion) |  | NGS  (425 panel) | Brain | SMARCA4(p. D1020del), TP53 (p.S127F), STAT3(p.H410R), PREX2(p.D114H), GATA3(p.P218H), NOTCH1(p. N605del), SDC4-ROS1 (fusion) |
| 19 | Dramatic progression | IHC | Pleura | ROS1(+) |  | RT-PCR | Lung | ROS1(+) |
| 20 | Dramatic progression | RT-PCR | Lymph nodes | ROS1(+) |  | Non-biopsy | Non-biopsy | Non-biopsy |
| 21 | Dramatic progression | NGS  (NA) | Lung | ROS1(+) |  | NGS  (NA) | Adrenal gland | ROS1(+) |
| 22 | Dramatic progression | NGS  (168 panel) | Lymph nodes | AURKA (amp), GNAS (amp), CD74-ROS1(fusion), MAST4-ROS1(fusion), ZNF217(p. T711=), ZNF217(p.Q584*), APC (p. E771fs) |  | Non-biopsy | Non-biopsy | Non-biopsy |
| 23 | Dramatic progression | IHC | Lymph nodes | ROS1(+) |  | Non-biopsy | Non-biopsy | Non-biopsy |
| 24 | Dramatic progression | IHC | Lung | ROS1(+) |  | Non-biopsy | Non-biopsy | Non-biopsy |
| 25 | Dramatic progression | RT-  PCR | Lymph nodes | ROS1(+) |  | Non-biopsy | Non-biopsy | Non-biopsy |
| 26 | Dramatic progression | NGS  (425 panel) | Lymph nodes | PTK2(c.2986-1G>A), KDR (p.P561A), TP53(p.P278S), SLC34A2-ROS1 |  | NGS  (425 panel) | Pleural effusion | ROS1(p.G2032R), NQO1(p.P187S), UGT1A1(p.G71R), PTK2(c.3013-1G>), SLC34A2-ROS1, TP53(p.P278S), DPYD (p.R29C), DPYD (p.I543V) |
| 27 | Dramatic progression | NGS  (139 panel) | Lymph nodes | ROS1(+) |  | NGS  (139 panel) | Lung | CD74-ROS1(fusion), ROS1（p.L2010M）, ROS1（KCNN2:5'UTR~ROS1:exon34）, RET（p.G830A)）, SMARCA4(p.R1192C), TP53(p.R213*), CDKN2A(p.R58*) |
| 28 | Dramatic progression | NA | Lymph nodes | ROS1(+) |  | NGS  (NA) | Chest nodule | NA |

Abbreviations: ROS1(+), c-ros oncogene 1 rearrangement positive; NGS, next generation sequencing, NA, not available, IHC, immunohistochemistry, RT-PCR, reverse transcription-polymerase chain reaction.
